# Microvascular Changes in Full-Thickness Macular Hole Patients Before and After Vitrectomy: An Optical Coherence Tomography–Angiography Study

**DOI:** 10.3390/clinpract15030058

**Published:** 2025-03-11

**Authors:** Aleksandra Górska, Sebastian Sirek, Dawid Woszczek, Rafał Leszczyński

**Affiliations:** 1 Department of Opthalmology, Faculty of Medical Sciences in Katowice, Medical University of Silesia, 40-752 Katowice, Poland; 2 Department of Opthalmology, Prof. K. Gibiński University Clinical Center, Medical University of Silesia in Katowice, 40-514 Katowice, Poland; 3 Students’ Scientific Society, Department of Ophthalmology, Faculty of Medical Sciences in Katowice, Medical University of Silesia, 40-752 Katowice, Poland

**Keywords:** full-thickness macular hole, vitrectomy, OCT–angiography, foveal avascular zone, vessel density, retinal microvasculature

## Abstract

**Background:** This study evaluates changes in the foveal avascular zone (FAZ) area and vessel density in patients with full-thickness macular hole (FTMH) before and after vitrectomy using optical coherence tomography–angiography (OCT-A). **Methods:** A prospective analysis was conducted on 39 patients diagnosed with FTMH who underwent pars plana vitrectomy. OCT-A imaging was performed preoperatively and at 1 and 3 months postoperatively. Parameters analyzed included FAZ area, superficial (SCP) and deep retinal vessel density (DCP), and central retinal thickness (CRT). **Results:** Preoperative OCT-A images showed a significant difference in the mean FAZ area between affected and healthy eyes (p < 0.01). However, a significant reduction in superficial capillary plexus (SCP) vessel density was noted after vitrectomy. Visual acuity improved significantly after surgery (p < 0.001), but no significant changes in FAZ or total vessel density were observed postoperatively. Postoperative distance visual acuity (DBCVA) correlated with preoperative macular hole size (p < 0.01). **Conclusions:** Vitrectomy for FTMH does not significantly alter the FAZ area or DCP vessel density, but does reduce SCP vessel density. OCT-A is a valuable tool for assessing retinal microvascular changes post-vitrectomy.

## 1. Introduction

Full-thickness macular hole (FTMH) is a significant cause of visual impairment, often requiring surgical intervention via pars plana vitrectomy (PPV). While PPV is effective in restoring retinal structure and improving visual acuity, its impact on retinal microvasculature, particularly the foveal avascular zone (FAZ) and vessel density, remains poorly understood. Optical coherence tomography–angiography (OCT-A) has emerged as a valuable tool for assessing retinal microvascular changes. This study aims to evaluate the effects of vitrectomy on FAZ area and vessel density in FTMH patients using OCT-A.

## 2. Materials and Methods

A total of 39 patients were included in the study, of whom 30 (77%) were women and 9 (23%) were men. All patients diagnosed with FTMH were scheduled to undergo PPV. The study was conducted at the Department of Ophthalmology, the University Clinical Center of Medical University of Silesia in Katowice, during the period between 1 January 2023 and 31 February 2024; all patients provided informed consent. Inclusion criteria were a clinical diagnosis of FTMH confirmed via spectral-domain optical coherence tomography (SD-OCT, Zeiss), age greater than 18 years old, and a willingness to comply with the study protocol. Exclusion criteria included previous retinal surgery, other retinal pathologies, or significant media opacities that could affect image quality, history of uveitis and retinitis, past trauma to the eye, high myopia (≥−6.0 Dsph), congenital or genetically determined retinal diseases, other eye diseases that interfere with retinal function (e.g., intraocular tumors, conditions after intraocular foreign body, conditions after disorders of the circulation in the retinal vessels), diseases of the anterior segment of the eye that prevent pupil dilation, retinal dystrophy or age-related macular degeneration, condition after anti-VEGF injections, epilepsy, connective tissue diseases, chronic oncological treatment, chronic immunosuppression, hyperbaric therapy, and lack of consent from the patient.

All patients underwent standard 23-gauge pars plana vitrectomy (PPV) performed by an experienced retinal surgeon. The surgery involved inducing posterior vitreous detachment, peeling the internal limiting membrane (ILM) using the standard inverted flap technique, and gas tamponade using sulfur hexafluoride (SF6). Postoperative face-down positioning was advised for 7 days to facilitate macular hole closure.

### 2.1. Imaging and Measurements

OCT-A was performed using Optovue, XR Avanti. Scans were obtained preoperatively, as well as at 1 month and 3 months postoperatively. Each scan covered a 6 × 6 mm area centered on the fovea. The foveal avascular zone (FAZ) area, macular hole minimal (MD) and base diameter (BD), central retinal thickness (CRT), and the parafoveal and perifoveal retinal vascular densities of the superficial capillary plexus (SCP) and deep capillary plexus (DCP) were measured and assessed using OCT-A. The distance (DBCVA) and near best-corrected visual acuity (NBCVA) were examined before surgery, as well as at 1 and 3 months after surgery.

### 2.2. Statistical Analysis

The normality of the distribution of the obtained results was assessed using the Shapiro–Wilk test and quantile–quantile (Q–Q) plots. For normally distributed data, the results were presented as mean ± standard deviation (M ± SD), while for non-normally distributed data, they were expressed as the median and interquartile range (Me (Q_1_–Q_3_)). For selected normally distributed data, the box plot included mean ± standard error, where the “whiskers” represented the 95% confidence interval (CI = 95%).

To compare data from two independent groups with normal or skewed distributions, Student’s *t*-test or the Mann–Whitney U test was used, respectively. For two dependent groups with a skewed distribution, the Wilcoxon signed-rank test was applied. Homogeneity of variance was assessed using the Fisher–Snedecor test. A comparison of multiple dependent samples with a normal distribution and sphericity assumption was performed using repeated-measures ANOVA, considering one or two factors. Sphericity was verified using Mauchley’s test. If the sphericity assumption was violated, the Greenhouse–Geisser correction was applied in the ANOVA test. For skewed data, the Friedman rank ANOVA test was used. To assess relationships and determine the strength of association between two variables with a skewed distribution, Spearman’s rank correlation test was applied.

A *p*-value < 0.05 was considered statistically significant, and all statistical tests were two-tailed.

Statistical analyses and figures were generated using Statistica version 13.

### 2.3. Ethical Considerations

This study was conducted in accordance with the principles outlined in the Declaration of Helsinki and received approval from the Bioethics Committee (nr. PCN/CBN/0052/KB1/122/22).

Written informed consent was obtained from all participants prior to their enrollment.

## 3. Results

The age of the female participants ranged from 51 to 78 years, with a mean ± standard deviation of 67.6 ± 5.6 years. The age of the male participants ranged from 59 to 74 years, with a mean ± standard deviation of 68.1 ± 5.13 years. The overall mean age of the study population was 67.9 ± 5.3 years. No statistically significant differences in age were observed between genders (*p* = 0.78). The hole was closed in all eyes after the initial surgery.

Significant differences in the visual acuity of DBCVA and NBCVA (logMAR) were found between the diseased eye group (MH group) and the fellow eye group (control group). At the same time, the values of DBCVA (logMAR) in the diseased eye group were higher than those of DBCVA (logMAR) in the control group, indicating worse visual acuity of the diseased eye to the far side compared with the healthy eye. On the other hand, the NBCVA (logMAR) values in the FTMH eye group were lower than the NBCVA (logMAR) values in the control group, indicating worse visual acuity of the affected eye for near compared to the healthy eye.

**Figure 1:**
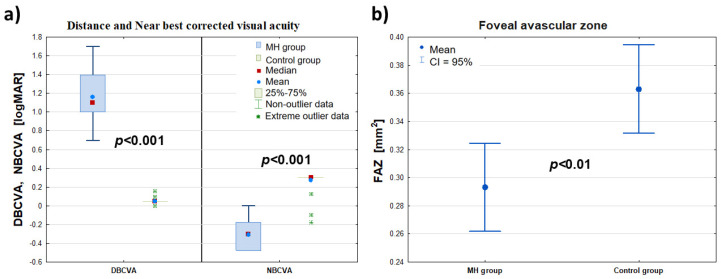
Comparison of the DBCVA, NBCVA (logMAR)—panel (**a**)—and FAZ (mm^2^)—panel (**b**)—of the affected eye (MH group) with the healthy eye (control group) before the procedure (at t_0_). The symbols (∎), (•), and (*) denote the median, mean, and extreme values, respectively; the ‘whiskers’ denote the range of outliers and the 95% confidence interval, respectively.

When the foveal area free zone (FAZ) was examined, there was a significant difference in the mean value of this parameter in the affected eye group compared to the fellow eye group, which differed by 0.07 mm^2^ (confidence interval 0.95: 0.114–0.026 mm^2^). At the same time, the mean FAZ area (mm^2^) in the FTMH eye group was smaller than in the healthy eye group. Detailed data are summarized in Table 1 and Figure 1.

There was a statistically significant difference between the values of the DBCVA (logMAR) parameter in the patient’s FTMH eye group studied before surgery, as well as at 1 and 3 months after surgery (*p* < 0.001). The median DBCVA (logMAR) in the patient’s FTMH eye group before surgery decreased from 1.097 to 0.398 at 3 months after surgery, indicating an improvement in the patient’s eye’s visual acuity to distance after the procedure.

The near visual acuity (NBCVA) test also showed a statistically significant difference between the (logMAR) values of this parameter for the patient’s FTMH eye group tested before surgery and at 1 and 3 months after surgery (*p* < 0.001). At the same time, the median NBCVA (logMAR) in the patient’s FTMH eye group increased from −0.301 before surgery to 0.301 3 months after surgery. Thus, the surgery performed improved the near visual acuity of the patient’s eye.

**Figure 2:**
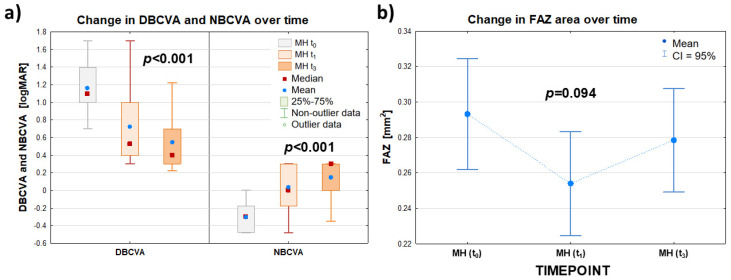
Summary data for best-corrected distance visual acuity (DBCVA) and near visual acuity (NBCVA) expressed in (logMAR)—panel (**a**)—and for foveal area free (FAZ) (mm^2^)—panel (**b**)—of the patient eye group (MH group) examined before surgery (t_0_) and at follow-up visits at 1 (t_1_) and 3 (t_3_) months after surgery. The symbols (∎) and (•) denote the median and mean value, respectively; the ‘whiskers’ denote the range of non-outliers—panel (**a**)—and the 95% confidence interval—panel (**b**)—respectively.

In contrast, the mean FAZ values determined for the patient’s eye before surgery, t_0_, and at the follow-up examinations 1 and 3 months after surgery, t_1_ and t_3_, were not statistically significantly different (*p* = 0.094). Thus, the surgery had no significant effect on changes in the values of this parameter. Detailed data are summarized in Table 2 and Figure 2.

**Figure 3:**
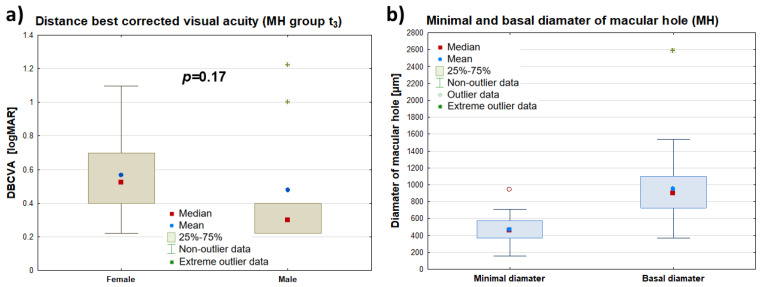
Comparison of DBCVA (logMAR) of the diseased eye measured 3 months postoperatively (MH t_3_) by patient gender—panel (**a**)—and a summary of data for the minimal and maximum macular hole diameters of the diseased eye (μm)—panel (**b**). The symbols (∎), (•), and (*) represent the median, mean value, and extreme values, respectively, while the “whiskers” denote the range of non-outlying values.

No statistically significant difference was found in the best-corrected distance visual acuity (DBCVA) (logMAR) of the diseased eye 3 months postoperatively (t_3_) between the female group (*n* = 30; Me(Q_1_–Q_3_): 0.3 (0.2–0.4)) and the male group (*n* = 9; Me(Q_1_–Q_3_): 0.5 (0.4–0.6)) (*p* = 0.17). The Me(Q_1_–Q_3_) of the minimal macular hole diameter (FTMH) was 465 (368–571) μm. Meanwhile, the Me(Q_1_–Q_3_) of the maximum macular hole diameter in the diseased eye was 902 (726–1100) μm. The maximum diameter was measured at the level of the retinal pigment epithelium (RPE), while the minimum diameter was measured at the narrowest point of the macular hole. Detailed data can be found in Figure 3.

The postoperative DBCVA (distance visual acuity) values of the FTMH eye had a statistically significant, high positive correlation with minimum (rs = 0.60; *p* < 0.001) and maximum hole diameter (rs = 0.50; *p* < 0.01). The postoperative DBCVA (logMAR) values of the FTMH eye decreased in relation to decreasing values of minimum and maximum hole diameters, indicating a worse distance visual acuity, the larger the diameters of these holes were before surgery.

In contrast, there was no statistically significant correlation of the postoperative DBCVA parameter of the FTMH eye with selected characteristics such as patient age, hole duration, CRT, time between surgery and first examination, FAZ parameter, or total retinal vascular plexus density SCP and DCP. Detailed data are summarized in Table 3.

The Me(Q_1_–Q_2_) value of the time between the patient’s first examination and surgery (PPV) was 11 (0–28) days, with a maximum time between examination and surgery of 119 days.

Analyzing each group separately, in the control group, no significant differences in SCP were found over time (*p*(t_0_ vs. t_1_) = 0.815; *p*(t_0_ vs. t_3_) = 0.807; and *p*(t_1_ vs. t_3_) = 0.652). In the MH group, a significant difference in the SCP of the affected eye was observed at 1 and 3 months postoperatively compared to the baseline SCP value before the surgery (*p*(t_0_ vs. t_1_) < 0.001 and *p*(t_0_ vs. t_3_) < 0.001, respectively). When comparing the mean SCP values at t_1_ and t_3_ (postoperative follow-up examinations), no significant differences were found (*p* = 0.273). The data are summarized in Table 4. The analysis shows that the full-thickness macular hole surgery reduced the total superficial retinal vascular plexus (SCP) density in the affected eye (MH) compared to the healthy eye.

On the other hand, statistical analysis performed for the relationship between the DCP values and group (MH/control group) independently of time, as well as for time (t_0_, t_1_, and t_3_) independently of group, showed no significant differences (*p* = 0.796 and *p* = 0.053, respectively). The data are presented in Figure 3 in panels c) and d). No significant interaction was found between the groups (MH/control group) and the examination time (t_0_, t_1_, and t_3_) (*p* = 0.747). The data from the DCP image analysis are summarized in Table 3. The analysis indicates that full-thickness macular hole surgery did not significantly affect the changes in the total density of the deep retinal vascular plexus (DCP image) in the affected eye (MH) compared to the healthy eye.

**Figure 4:**
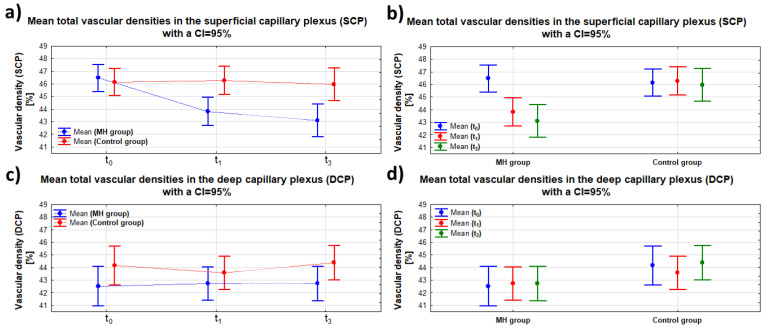
Dependence of SCP and DCP on time (t_0_, t_1_, t_3_)—panel (**a**,**c**)—and on group (MH/control group)—panel (**b**,**d**)—with the same scale span; “whiskers” indicate the 95% confidence interval.

Detailed data on the relationship between SCP and DCP parameters with respect to time and group are presented in Figure 4a–d.

The density of the superficial retinal vascular plexus in the parafovea, parafovea superior, parafovea nasal, parafovea inferior, and parafovea temporal areas in the diseased eye group (MH group) before surgery (at t_0_) did not differ significantly from the healthy eye group (control group), respectively, at *p* = 0.80, *p* = 0.66, *p* = 0.13, *p* = 0.28, and *p* = 0.29. However, there was a significant difference in vascular density in the SCP–fovea area in the diseased eye group compared to the healthy eye group (*p* < 0.001), with mean vascular density values in this area being higher in the diseased eye group. For deep choroid plexus density measurements, there were significant differences in vessel density in the parafovea, parafovea superior, and parafovea temporal areas in the diseased eye compared to the healthy eye (*p* < 0.05, *p* < 0.001, and *p* < 0.05, respectively), with lower values in the diseased eye group. In the DCP–fovea area, there was also a significant difference in retinal choroid plexus density in the diseased eye compared to the healthy eye (*p* < 0.05), but the values were smaller in the healthy eye group. In the DCP areas of the parafovea nasal and parafovea inferior studied, there were no significant differences in mean vascular density between the diseased and healthy eye (*p* = 0.072 and *p* = 0.25, respectively). The analysis shows that the density of the superficial retinal choroid plexus (SCP) for all PARA areas of the diseased eye was not significantly different from that of the healthy eye. The opposite is true for the DCP, for which significant differences in vascular density were found in most of the DCP areas of the diseased eye examined, i.e., fovea, parafovea, superior, temporal) compared to the healthy eye.

In both SCP-PERI and DCP-PERI image analysis, no significant differences were found between the retinal vascular plexus density of the diseased and healthy eye, respectively, in the areas of perifovea (*p* = 0.97; *p* = 0.11), perifovea superior (*p* = 0.66; *p* = 0.13), perifovea nasal (*p* = 0.71; *p* = 0.12), perifovea inferior (*p* = 0.96; *p* = 0.19), and perifovea temporal (*p* = 0.99; *p* = 0.25), respectively. Detailed data are summarized in Table 5.

The density of the superficial retinal vascular plexus in the parafovea, superior parafovea, nasal parafovea, inferior parafovea, and temporal parafovea areas of the patient’s eye (MH) before the procedure differed significantly from the vessel density after the procedure, at *p* < 0.001, *p* < 0.001, *p* < 0.05, *p* < 0.001, and *p* < 0.001, respectively. However, vessel densities in these areas decreased after the procedure. In contrast, the treatment did not significantly affect vessel density in the fovea area (*p* = 0.588). When measuring the density of the DCP, no significant changes in vascular density as a result of the surgery were shown in the areas studied.

The density of the superficial retinal vascular plexus in the diseased eye group before full-thickness macular hole surgery was significantly different from the vessel density after surgery in all of the following areas: perifovea (PERI), perifovea superior, perifovea nasal, perifovea inferior, and perifovea temporal, respectively, at *p* < 0.001, *p* < 0.001, *p* < 0.001, *p* < 0.001, and *p* < 0.05. At the same time, vascular densities in these areas decreased after the procedure. When the images of the DCP-PERI areas were analyzed, there was no significant effect of the treatment on changes in vascular densities in any of the areas. Detailed data are summarized in Table 6.

The analysis shows that the treatment significantly affected only the retinal choroid plexus density of the parafovea (PARA) and PERI areas of the SCP image. In contrast, for the DCP image, the treatment did not significantly affect the density of the retinal choroid plexus in the PARA and PERI areas considered.

The strength of the correlation between the retinal choroid plexus density of the patient’s eye before surgery (t_0_), in each of the areas studied, as well as the DBCVA parameter of the patient’s eye studied 3 months after surgery (t_3_), was weak and not statistically significant. Detailed data are summarized in Table 7.

The CRT parameter values determined for the diseased eye were significantly different compared to the healthy eye (*p* < 0.001). At the same time, CRT values in the diseased eye group were higher compared to the healthy eye group. There were also significant differences between the CRT values of the affected eye before and 3 months after surgery (*p* < 0.001). CRT values in the diseased eye group after surgery (examined at t_3_) were not statistically significantly different compared to the healthy eye group (*p* = 0.111). Table 8.

## 4. Discussion

In our study, no statistically significant correlation was found between postoperative DBCVA and macular hole duration. However, a strong correlation was observed between postoperative DBCVA and the minimum hole diameter, suggesting that anatomical factors may play a more significant role in visual recovery than symptom duration. Interestingly, Wong et al. and Ryan et al. reported that macular hole surgery tends to yield better visual outcomes in patients with symptoms lasting less than six months. This discrepancy may stem from differences in study populations, surgical techniques, or the complex interplay between macular hole duration and retinal microstructural changes. Further research is needed to clarify the relative impact of these factors on postoperative visual acuity [1,2].

Our outcomes are comparable to the study by Lamas-Francis, which focused specifically on macular holes greater than 400 μm in diameter. Both studies highlight the importance of hole size in predicting surgical outcomes. In particular, Lamas-Francis demonstrated that the inverted flap technique (IFT) offers a high rate of anatomical and functional recovery, even for larger macular holes. Similarly, our findings suggest that macular hole diameter plays a crucial role in determining the success of surgery, with larger holes requiring careful assessment and management. The positive outcomes seen in both studies emphasize the potential for improved visual recovery even in cases involving larger macular holes, reinforcing the significance of precise preoperative evaluation and tailored surgical approaches [3].

In addition to these findings, it is important to note that retinal healing does not stop immediately after surgery. Macular holes close with a bridge of glial cells and over time, the elevation in the outer retinal layers—often observed as a slight bulging or irregularity on OCT imaging—gradually flattens and normalizes. This reduction in elevation reflects the progressive realignment and reattachment of the photoreceptor layer and other outer retinal structures. Interestingly, photoreceptor defects continue to shrink for up to 12 months following surgery, and this ongoing improvement is closely tied to better visual acuity. These longer-term changes suggest that while early postoperative results are important, true visual recovery may rely on the continued healing of the retina. This underscores the idea that alongside the duration of symptoms, the ongoing process of retinal regeneration plays a key role in determining long-term visual outcomes. [4].

In contrast to some previous studies reporting a reduction in the FAZ area post-vitrectomy, our findings indicate that the procedure did not significantly affect this parameter. Michalewska et al. (2010) [4] suggested that successful macular hole closure leads to a decrease in the FAZ area due to the restoration of normal retinal architecture and perfusion. However, our results do not corroborate these findings, as we observed no statistically significant changes in FAZ values in the diseased eye before surgery (t_0_) and at follow-up examinations 1 and 3 months postoperatively. This suggests that vitrectomy may not have a substantial impact on FAZ area changes in this context [5]. Similarly, Kim et al. (2018) [6] reported minimal changes in FAZ area after vitrectomy for macular holes, which aligns with our findings. However, other studies, such as those by Spaide et al. (2015) [7] and Michalewska et al. (2008) [5], demonstrated a reduction in FAZ area following vitrectomy, particularly in patients with epiretinal membranes. This discrepancy may be attributed to differences in study design, patient populations, or the timing of postoperative assessments [5,7].

Similarly, Kita et al. also performed a 1-month follow-up and observed a significant reduction in FAZ areas postoperatively. While Kita’s study suggests a more immediate effect of macular hole surgery on FAZ area, our results imply that such changes may not be as prominent within the first few months after surgery. These discrepancies may be due to differences in study protocols, such as variations in the assessment methods or the specific patient populations. It is also possible that the dynamics of retinal recovery following surgery may vary between individuals, with some showing quicker changes in the FAZ area than others [8].

In Ando’s study, a significant reduction in the FAZ area was observed from 0.42 ± 0.08 mm^2^ preoperatively to 0.24 ± 0.07 mm^2^ at 1 month postoperatively, with a slight increase to 0.25 ± 0.06 mm^2^ at 12 months postoperatively. In our study, while the FAZ area showed no statistically significant changes at 1 and 3 months postoperatively, we did observe a slight increase in the FAZ area at the 3-month follow-up. However, this increase did not reach statistical significance (*p* = 0.094) [9].

Additionally, although Ando et al. found a significant correlation between the FAZ ratio and visual acuity at 6 and 12 months, we did not observe a similar relationship in our study. This difference may be attributed to the shorter follow-up duration in our research (1 and 3 months postoperatively), while Ando et al. extended their follow-up to 12 months. As the process of retinal remodeling and recovery may continue beyond the 3-month window, the follow-up period in our study may not have been long enough to capture more pronounced or statistically significant changes in the FAZ that could be related to long-term visual outcomes [9].

Our findings suggest that FTMH surgery leads to a reduction in the total density of the SCP in the diseased eye compared to the healthy eye, while no significant changes were observed in the DCP. This contrasts with some previous studies, such as that of Teng et al. [10], which reported an increase in retinal vessel density post-macular hole surgery, likely due to the re-establishment of the vitreoretinal interface and the removal of tractional forces. However, our results indicate that while vitrectomy may alter SCP density, it does not significantly impact DCP or FAZ area. These findings are supported by Kim et al. [6], who also reported a decrease in SCP density after vitrectomy, suggesting that the superficial retinal layers may be more susceptible to surgical manipulation [11].

Our results partially align with the findings of Teng et al. [10], who reported that parafoveal vessel density in eyes with FTMH was significantly lower than in healthy control eyes. Similarly, in our study, we observed reduced vascular density in the SCP of the FTMH eyes compared to the fellow eyes, particularly in the foveal area. However, there are notable differences between our findings and those of Teng et al. [10] in the postoperative course. Teng et al. [10] reported a significant increase in parafoveal vessel density one month after vitrectomy, whereas our study did not show significant changes in DCP vascular density at the 1- and 3-month follow-up. This discrepancy may be attributed to differences in the timing of postoperative assessments, patient characteristics, and the specific methodologies used to analyze vascular changes [10].

Our findings regarding the changes in SCP and DCP post-vitrectomy align partially with some of the results from Hwang et al. In their study, they observed a significant increase in SCP vessel density in the center area six months postoperatively (*p* = 0.003), while the DCP vessel density did not show significant changes compared to the preoperative state. In contrast, our study, with follow-up at 1 and 3 months postoperatively, did not show significant changes in either SCP or DCP vessel densities. These differences may suggest that longer observation periods might be necessary to fully capture the remodeling of retinal vascular structures following macular hole surgery [12].

When comparing our findings with the results of Yun et al., there are notable differences in the observed effects on retinal vascular parameters after macular hole surgery. Yun et al. reported a reduction in both the SCP and DCP vascular densities in the macular hole group compared to controls, with significant differences in both SCP (*p* = 0.019) and DCP (*p* = 0.003). In contrast, our study showed that while there was a significant reduction in the SCP in the affected eye at 1 and 3 months post-surgery (*p* < 0.001), no significant differences were observed between follow-up timepoints (*p* = 0.273). Additionally, we found no significant changes in the DCP densities post-surgery (*p* = 0.796), indicating a lack of significant remodeling in the deep retinal vasculature in our cohort [13]. The observed reduction in superficial capillary plexus (SCP) vessel density following vitrectomy may be attributed to several factors, including phototoxicity from endoillumination during surgery, which could damage the retinal microvasculature, particularly in the superficial layers. Additionally, the use of sulfur hexafluoride (SF6) gas as a tamponade agent may induce mechanical stress on the retinal vessels, particularly in the superficial layers, leading to temporary or permanent changes in vessel density.

It is also noteworthy that OCT-A has proven to be a valuable tool in monitoring these microvascular changes. Its non-invasive nature and ability to provide high-resolution images of retinal vasculature make it an essential modality in both clinical and research settings. As Spaide et al. (2015) pointed out, OCT-A offers unique insights into the retinal microcirculation that are not possible with traditional imaging techniques [14].

### Limitations and Future Directions

While our study provides significant insights into the microvascular changes following vitrectomy for FTMH, it is not without limitations. The relatively small sample size and short follow-up period may limit the generalizability of our findings. Future studies with larger cohorts and longer follow-up periods are needed to validate our results. Additionally, exploring the impact of different surgical techniques and tamponade agents on microvascular outcomes could provide further valuable information.

## 5. Conclusions

In conclusion, this study demonstrates that vitrectomy for FTMH does not significantly alter the FAZ area or the total vessel density of the DCP. However, a reduction in the total vessel density of the SCP was observed postoperatively in the FTMH eye compared to the fellow eye. Additionally, no statistically significant correlation was found between postoperative distance visual acuity (DBCVA) and SCP or DCP, FAZ parameters, patient age, macular hole duration, or central retinal thickness (CRT).

These findings suggest that while vitrectomy successfully restores retinal structure, its effects on retinal microvasculature may be layer-specific and do not directly influence visual acuity recovery. Moreover, the absence of significant FAZ changes contrasts with previous reports, indicating the need for further research to understand the complex relationship between retinal perfusion, microvascular changes, and functional outcomes.

## Figures and Tables

**Table 1:** Distance best-corrected visual acuity (DBCVA) (logMAR) and near best-corrected visual acuity (NBCVA) (logMAR), as well as fovea avascular zone—FAZ (mm^2^)—of preoperative eyes with full-thickness macular hole (FTMH group) and fellow eyes (control group).

*n* = 39	FTMH Group (t_0_)	Fellow Eye (t_0_)	*p*-Value
DBCVA (logMAR)	1.097 (1.00–1.398)	0.046 (0.046–0.046)	<0.001
NBCVA (logMAR)	−0.301 (−0.478–−0.176)	0.301 (0.301–0.301)	<0.001
FAZ (mm^2^)	0.293 ± 0.097	0.363 ± 0.097	<0.01

M ± SD—mean ± standard deviation; Me (Q_1_–Q_3_)—median (lower–upper quartile); *p*—statistical significance.

**Table 2:** Distance best-corrected visual acuity (DBCVA) and near best-corrected visual acuity (NBCVA) (logMAR), as well as fovea avascular zone—FAZ (mm^2^)—of eyes with macular hole (MH group) at preoperative (t_0_) and postoperative (t_1_, t_3_) timepoints.

*n* = 39	TIMEPOINT			*p*-Value
	MH Group (t_0_)	MH Group (t_1_)	MH Group (t_3_)	
DBCVA (logMAR)	1.097 (1.000–1.398)	0.523 (0.398–1.000)	0.398 (0.301–0.699)	<0.001
NBCVA (logMAR)	−0.301 (−0.478–−0.176)	0.00 (−0.176–0.301)	0.301 (0.00–0.301)	<0.001
FAZ (mm^2^)	0.29 ± 0.097	0.254 ± 0.090	0.278 ± 0.090	0.094

M ± SD—mean ± standard deviation; Me (Q_1_–Q_2_)—median (lower–upper quartile); *p*—statistical significance.

**Table 3:** Measures of the correlation of the DBCVA (logMAR) trait of the patient’s eye after the procedure (follow-up 3 months after the procedure, t_3_) in relation to selected traits of the patient’s eye before the procedure (t_0_).

Selected Parameters, MH Group (t_0_)		*r_s_*	*p*-Value
DBCVA HM group (t_3_) (logMAR)	MH diameter (minimal) (μm)	0.60	<0.001
	MH diameter (basal) (μm)	0.50	<0.01
	Age (years)	0.28	0.083
	Time between the patient’s first examination and PPV (months)	0.22	0.176
	Central retinal thickness (μm)	−0.22	0.180
	Time between PPV and examination before PPV (days)	0.13	0.447
	FAZ (mm^2^)	0.10	0.547
	Whole vascular density SCP (%)	0.04	0.832
	Whole vascular density DCP (%)	−0.15	0.351

**Table 4:** Mean total vascular densities in the superficial capillary plexus (SCP) and deep capillary plexus (DCP) of the diseased eye (MH group) compared to the healthy eye (control group), measured before the surgery (t_0_) and during follow-up visits at 1 month (t_1_) and 3 months (t_3_) postoperatively.

SCP (%)	***n* = 39**	**t_0_**	**t_1_**	**t_3_**	***p*-Value (Independent of MH and Control Groups)**	***p*-Value (Independent of Timepoints (t_0_, t_1_, t_3_))**	***p*-Value (Interaction Between Groups and Timepoints)**
	MH group	46.48 ± 2.93	43.83 ± 3.31	43.10 ± 4.49	<0.001	<0.05	<0.001
	Control group	46.15 ± 3.74	46.28 ± 3.71	45.98 ± 3.60			
	*p*-value	0.660	<0.01	<0.01			
DCP (%)	***n* = 39**	**t_0_**	**t_1_**	**t_3_**	***p*-Value (Independent of MH and Control Groups)**	***p*-Value (Independent of Timepoints (t_0_, t_1_, t_3_))**	***p*-Value (Interaction Between Groups and Timepoints)**
	MH group	42.53 ± 4.69	42.74 ± 4.43	42.74 ± 4.58	0.796	0.053	0.747
	Control group	44.18 ± 5.02	43.57 ± 3.81	44.41 ± 3.98			
	*p*-value	0.138	0.375	0.091			

**Table 5:** Comparison of preoperative vascular density (%) of eyes between the macular hole (MH) group (*n* = 39) and control group (*n* = 39) at different sectors.

Vascular Density (%)	MH Group (*n* = 39)	Control Group (*n* = 39)	*p*-Value
**SCP**			
FOVEA	23.31 ± 7.37	15.68 ± 6.23	<0.001
PARAFOVEA	48.65 ± 3.96	48.42 ± 4.05	0.80
PARAFOVEA SUPERIOR	49.00 ± 5.21	49.55 ± 5.56	0.66
PARAFOVEA NASAL	48.35 (46.3–50.5)	45.30 (41.4–50.2)	0.13
PARAFOVEA INFERIOR	48.92 ± 5.65	47.46 ± 6.06	0.28
PARAFOVEA TEMPORAL	49.75 ± 4.43	48.53 ± 5.80	0.29
**DCP**			
FOVEA	32.85 ± 6.02	29.05 ± 6.16	<0.05
PARAFOVEA	48.63 ± 4.66	51.11 ± 4.84	<0.05
PARAFOVEA SUPERIOR	46.23 ± 6.12	51.58 ± 5.47	<0.001
PARAFOVEA NASAL	49.4 (47.0–52.2)	52.9 (48.8–56.7)	0.07
PARAFOVEA INFERIOR	47.4 (44.2–50.1)	49.0 (44.7–53.1)	0.25
PARAFOVEA TEMPORAL	49.5 (47.4–53.3)	52.1 (49.5–56.4)	<0.05
**Vascular Density (%)**	**MH Group (*n* = 39)**	**Control Group (*n* = 39)**	***p*-Value**
**SCP**			
PERIFOVEA	46.78 ± 3.48	46.82 ± 4.06	0.97
PERIFOVEA SUPERIOR	46.48 ± 3.72	46.88 ± 4.32	0.66
PERIFOVEA NASAL	50.81 ± 3.28	50.51 ± 3.80	0.71
PERIFOVEA INFERIOR	47.14 ± 4.64	47.09 ± 4.64	0.96
PERIFOVEA TEMPORAL	42.74 ± 4.53	42.76 ± 5.24	0.99
**DCP**			
PERIFOVEA	42.88 ± 5.17	44.86 ± 5.71	0.11
PERIFOVEA SUPERIOR	41.31 ± 6.67	43.69 ± 7.26	0.13
PERIFOVEA NASAL	41.42 ± 6.85	43.80 ± 6.50	0.12
PERIFOVEA INFERIOR	41.40 ± 6.21	43.31 ± 6.55	0.19
PERIFOVEA TEMPORAL	47.28 ± 4.38	48.60 ± 5.63	0.25

M ± SD—mean ± standard deviation; Me (Q_1_–Q_3_)—median (lower–upper quartile); *p*—statistical significance.

**Table 6:** Comparison of tomographic features between the preoperative MH 0 (*n* = 39) and postoperative MH 1 and MH 3 (1 and 3 months) (*n* = 39).

Vascular Density (%)	MH (*n* = 39)	MH 1 (*n* = 39)	MH 3 (*n* = 39)	*p*-Value
**SCP**				
FOVEA	23.31 ± 7.37	24.55 ± 6.83	23.27 ± 7.63	0.588
PARAFOVEA	48.65 ± 3.96	44.81 ± 3.60	43.64 ± 5.49	<0.001
PARAFOVEA SUPERIOR	49.00 ± 5.21	45.41 ± 4.81	44.78 ± 5.91	<0.001
PARAFOVEA NASAL	48.3 (45.3–50.5)	45.5 (40.8–47.5)	43.5 (38.9–47.8)	<0.05
PARAFOVEA INFERIOR	48.92 ± 5.65	45.63 ± 4.07	43.65 ± 6.94	<0.001
PARAFOVEA TEMPORAL	49.75 ± 4.43	44.78 ± 3.87	43.52 ± 4.46	<0.001
**DCP**				
FOVEA	32.85 ± 6.02	35.00 ± 6.51	33.52 ± 6.65	0.17
PARAFOVEA	48.63 ± 4.66	48.26 ± 4.46	47.77 ± 5.03	0.67
PARAFOVEA SUPERIOR	46.1 (42.8–50.5)	46.0 (42.8–49.7)	47.5 (45.3–50.6)	0.78
PARAFOVEA NASAL	50.09 ± 6.07	50.37 ± 4.60	49.30 ± 5.27	0.60
PARAFOVEA INFERIOR	47.67 ± 5.27	46.29 ± 5.61	46.30 ± 5.77	0.41
PARAFOVEA TEMPORAL	49.5 (47.4–53.3)	49.6 (47.7–53.2)	50.5 (48.0–53.2)	0.87
**Vascular Density (%)**	**MH (*n* = 39)**	**MH 1 (*n* = 39)**	**MH 3 (*n* = 39)**	***p*-Value**
**SCP**				
PERIFOVEA	46.78 ± 3.48	44.26 ± 3.73	43.22 ± 3.83	<0.001
PERIFOVEA SUPERIOR	46.48 ± 3.72	44.12 ± 4.72	42.84 ± 4.20	<0.001
PERIFOVEA NASAL	50.81 ± 3.28	48.33 ± 4.08	47.98 ± 4.51	<0.001
PERIFOVEA INFERIOR	47.14 ± 4.64	43.65 ± 4.23	43.17 ± 5.63	<0.001
PERIFOVEA TEMPORAL	42.74 ± 4.53	41.03 ± 4.08	39.63 ± 4.26	<0.05
**DCP**				
PERIFOVEA	42.88 ± 5.17	43.54 ± 4.91	43.64 ± 5.15	0.73
PERIFOVEA SUPERIOR	41.31 ± 6.67	42.62 ± 5.57	42.57 ± 6.03	0.46
PERIFOVEA NASAL	41.42 ± 6.85	43.01 ± 6.14	43.68 ± 6.55	0.22
PERIFOVEA INFERIOR	41.40 ± 6.21	41.82 ± 5.27	41.67 ± 6.33	0.95
PERIFOVEA TEMPORAL	47.28 ± 4.38	47.10 ± 5.48	46.80 ± 5.12	0.89

M ± SD—mean ± standard deviation; Me (Q_1_–Q_3_)—median (lower–upper quartile); *p*—statistical significance.

**Table 7:** Measures of the correlation of retinal vascular plexus density tested in different areas of the patient’s eye after the procedure performed (follow-up 3 months after the procedure (t_3_) in relation to the DBCVA (logMAR) of the patient’s eye before the procedure performed (t_0_)).

Vascular Density (%) (MH t_0_) (*n* = 39)	DBCVA (MH t_3_) (logMAR) (*n* = 39)	
	r_s_	*p*-Value
**SCP**		
FOVEA	−0.11	0.50
PARAFOVEA	0.04	0.79
PARAFOVEA SUPERIOR	0.06	0.73
PARAFOVEA NASAL	−0.03	0.88
PARAFOVEA INFERIOR	0.13	0.43
PARAFOVEA TEMPORAL	−0.12	0.48
**DCP**		
FOVEA	−0.1	0.55
PARAFOVEA	0.003	0.99
PARAFOVEA SUPERIOR	−0.12	0.45
PARAFOVEA NASAL	0.07	0.69
PARAFOVEA INFERIOR	0.08	0.65
PARAFOVEA TEMPORAL	−0.04	0.81
**Vascular Density (%) (MH t_0_) (*n* = 39)**	**DBCVA (MH t_3_) (logMAR) (*n* = 39)**	
	**r_s_**	***p*-Value**
**SCP**		
PERIFOVEA	−0.03	0.88
PERIFOVEA SUPERIOR	0.10	0.55
PERIFOVEA NASAL	−0.18	0.28
PERIFOVEA INFERIOR	−0.01	0.96
PERIFOVEA TEMPORAL	−0.11	0.50
**DCP**		
PERIFOVEA	−0.24	0.14
PERIFOVEA SUPERIOR	−0.25	0.13
PERIFOVEA NASAL	−0.21	0.39
PERIFOVEA INFERIOR	−0.14	0.45
PERIFOVEA TEMPORAL	−0.12	0.20

**Table 8:** Comparison of CRT (μm) between MH and control eyes, as well as MH preoperative and postoperative (at t_0_ vs. t_3_ timepoint).

*n* = 39	MH Group (t_0_)	Control Group (t_0_)	*p*-Value
**CRT** (μm)	291 (284–308)	283 (269–290)	<0.001
***n* = 39**	**MH Group (t_0_)**	**MH Group (t_3_)**	
**CRT** (μm)	291 (284–308)	288 (274–295)	<0.001
***p*-value**		0.111	

Me (Q_1_–Q_3_)—median (lower–upper quartile); *p*—statistical significance.

## Data Availability

The original contributions presented in this study are included in the article. Further inquiries can be directed to the corresponding author.
